# Nitrogen-Driven Orchestration of Lateral Root Development: Molecular Mechanisms and Systemic Integration

**DOI:** 10.3390/biology14081099

**Published:** 2025-08-21

**Authors:** Xichao Sun, Yingchen Gu, Yingqi Liu, Zheng Liu, Peng Wang

**Affiliations:** 1 Agro-Environmental Protection Institute, Ministry of Agriculture and Rural Affairs, Tianjin 300191, China; sunxichao@caas.cn; 2 Key Laboratory of Tobacco Biology and Processing, Ministry of Agriculture and Rural Affairs, Tobacco Research Institute, Chinese Academy of Agricultural Sciences, Qingdao 266101, China; gyczc201@163.com (Y.G.); 18769706718@163.com (Y.L.); 3 Institute of Crop Sciences, Chinese Academy of Agricultural Sciences, Beijing 100081, China; 4 Key Laboratory of Crop Physiology and Ecology, Ministry of Agriculture and Rural Affairs, Beijing 100081, China

**Keywords:** nitrogen signaling, lateral root development, nitrate transporter, auxin, transcription factors, systemic signaling, hormone crosstalk, nutrient sensing, root architecture plasticity

## Abstract

N, as plants’ most essential nutrient, profoundly shapes root system architecture (RSA), with LRs being preferentially regulated. This review synthesizes the intricate molecular mechanisms underpinning N sensing, signaling, and its integration into developmental pathways governing LR initiation, primordium formation, emergence, and elongation. We delve deeply into the roles of specific transporters (NRT1.1, nitrate transporter 2.1 (NRT2.1)), transcription factors (*Arabidopsis* nitrate regulated 1 (ANR1), NLP7, TGACG motif-binding factor (TGA), squamosa promoter-binding protein-like 9 (SPL9)) and intricate hormone signaling networks (auxin, abscisic acid, cytokinins, ethylene) modulated by varying N availability (deficiency, sufficiency, excess) and chemical forms (NO_3_^−^, NH_4_^+^, organic N). Emphasis is placed on the systemic signaling pathways, including peptide-mediated long-distance communication (CEP—C-terminally encoded peptide receptor 1 (CEPR1)) and the critical role of the shoot in modulating root responses. Furthermore, we explore the emerging significance of carbon–nitrogen (C/N) balance, post-translational modifications (ubiquitination, phosphorylation), epigenetic regulation, and the complex interplay with other nutrients (phosphorus (P), sulfur (S)) and environmental factors in shaping N-dependent LR plasticity. Recent advances utilizing single-cell transcriptomics and advanced imaging reveal unprecedented cellular heterogeneity in LR responses to N. Understanding this sophisticated regulatory network is paramount for developing strategies to enhance nitrogen use efficiency (NUE) in crops. This synthesis underscores how N acts as a master regulator, dynamically rewiring developmental programs through molecular hubs that synchronize nutrient sensing with root morphogenesis—a key adaptive strategy for resource acquisition in heterogeneous soils.

## Simple Summary

Nitrogen (N), as a vital nutrient, intricately regulates plant root architecture, particularly lateral root (LR) development. This review highlights the dual role of N as both a signaling molecule and structural component, influencing LR initiation, growth, and patterning through molecular hubs like nitrate transporter 1.1 (NRT1.1) transporters and NIN-like protein 7 (NLP7) transcription factors. Local N availability reshapes auxin gradients and hormone crosstalk (e.g., cytokinins, ethylene), while systemic signals via C-terminally encoded peptide (CEP) coordinate shoot–root communication. Advanced imaging reveals cell-type-specific N responses, underscoring the adaptive plasticity of root systems. Understanding this regulatory network is critical for breeding crops with optimized N uptake efficiency, addressing global sustainability challenges in agriculture.

## 1. Introduction

RSA is a dynamic and plastic trait fundamental to plant fitness, enabling efficient exploration and exploitation of heterogeneous soil environments for essential resources, particularly water and mineral nutrients [1,2]. Among the macronutrients, N stands paramount, frequently acting as the primary limiting factor for plant growth and agricultural productivity [3,4]. The plasticity of RSA in response to N availability represents a key adaptive strategy, allowing plants to optimize N acquisition by modulating root growth and branching patterns. While RSA includes primary roots, root hairs, and adventitious roots, this review focuses specifically on LRs as the primary drivers of RSA plasticity in response to N. LRs differ from primary roots in their post-embryonic origin (pericycle cells) and their exceptional sensitivity to local and systemic N signals—attributes that make them critical for optimizing N foraging in heterogeneous soils [5,6,7].

LR development is a highly coordinated morphogenetic program, initiated when pericycle founder cells undergo asymmetric division. This process is orchestrated by auxin gradients and involves precise transcriptional reprogramming. The pericycle cells destined to form LRs transition through distinct developmental checkpoints: (i) specification of founder cells, (ii) anticlinal and periclinal divisions forming a dome-shaped primordium, (iii) organization into distinct cell layers mimicking the primary root apical meristem, (iv) emergence through overlaying tissues via enzymatic loosening of cell walls and programmed cell death in endodermal barriers, and (v) activation of elongation and meristem establishment [8,9,10]. Each stage presents potential regulatory checkpoints influenced by N status.

LR development is modulated by a complex interplay of intrinsic and extrinsic factors. Intrinsically, developmental cues such as auxin gradients and cell cycle progression dictate LR initiation and growth. Extrinsically, environmental factors including nutrient availability (e.g., N, P, S), water status, soil pH, and microbial interactions shape LR plasticity. Additionally, systemic signals from the shoot, such as cytokinins and CEPs, coordinate LR development with whole-plant nutrient status and photosynthetic capacity. These factors converge on core regulatory modules (e.g., NRT1.1-NLP7, auxin response factor (ARF)–lateral organ boundaries domain (LBD)) to fine-tune LR patterning, ensuring adaptive root foraging in heterogeneous environments.

While the stimulatory effect of localized nitrate patches on LR elongation is well-documented [11,12,13], the molecular mechanisms governing how plants perceive varying N availability (deficiency, sufficiency, toxicity), interpret N form (nitrate, ammonium, amino acids), and transduce these signals into precise developmental reprogramming of LR formation across different stages are remarkably intricate and involve a sophisticated interplay of transporters, sensors, transcription factors, hormone signaling pathways, and systemic communication. This complexity arises because N signals must be integrated with endogenous developmental cues at spatial scales ranging from subcellular compartments (e.g., nuclear translocation of transcription factors) to whole-organism systemic communication, while simultaneously incorporating metabolic feedback from carbon fixation and energy status.

This review aims to provide a comprehensive and mechanistic synthesis of our current understanding of how N signaling pathways, operating at both local and systemic levels, are integrated with the core developmental machinery to exert exquisite control over LR development, thereby optimizing root foraging capacity in fluctuating N environments. We highlight recent conceptual advances, unresolved questions, and the implications for enhancing NUE in agriculture.

## 2. N-Sensing and Primary Signaling Modules in Roots

### 2.1. Nitrate Sensing: Transceptors and Transcriptional Hubs

The perception of N status, particularly nitrate, initiates a cascade of signaling events that profoundly influence gene expression and developmental processes. Central to this perception are specific transporters functioning dualistically as transceptors (transporters/receptors).

NRT1.1 represents the archetypal example. Beyond its high-affinity NO_3_^−^ transport capability at low external concentrations, NRT1.1 acts as a critical nitrate sensor [14,15]. Its phosphorylation status, dynamically regulated by calcineurin B-like protein-interacting protein kinase 23 (CIPK23) and calcineurin B-like protein 1/9 (CBL1/9) complexes in response to NO_3_^−^ availability, dictates its affinity state and signaling output [14,16]. Structural studies reveal that phosphorylation at threonine 101 (Thr101) induces a conformational shift in dimeric structure of NRT1.1, exposing a high-affinity nitrate-binding site while simultaneously unmasking signaling domains that interact with downstream partners [17]. This dual functionality allows NRT1.1 to transduce extracellular nitrate concentration gradients into intracellular biochemical signals. Phosphorylation of NRT1.1 at Thr101 by CIPK23 switches it to a high-affinity state under low NO_3_^−^, simultaneously triggering downstream signaling events independent of its transport function [15,17]. This signaling involves direct interaction and modulation of key transcription factors.

Crucially, NRT1.1 interacts with and inhibits the nuclear retention of NLP7, a master transcriptional regulator of primary nitrate responses [18,19]. Upon NO_3_^−^ perception, especially at higher concentrations, NLP7 is rapidly phosphorylated (potentially by calcium-dependent protein kinases like CPK10/30/32 downstream of NRT1.1-induced calcium influx) and translocates to the nucleus, where it binds to nitrate-responsive elements (NREs) in promoters of hundreds of NO_3_^−^-responsive genes, including those involved in N assimilation and transport [20,21,22]. The NRE motif serves as a genomic docking site for NLP7 homodimers, which recruit chromatin remodeling complexes to activate transcription. This process is further fine-tuned by the oligomerization state of NLP7 and interaction with coregulators like hypersensitive to low P and low nitrate response 1 (HRS1) [23]. The phosphorylation status of NLP7, regulated by phosphatases like long-chain base 2 (LCB2), is critical for its activity and nuclear localization [24].

Another essential transcription factor family regulated by NO_3_^−^ sensing is TGA1 and TGA4. These basic leucine zipper (bZIP) transcription factors form complexes with NLP7 to activate transcription of key NO_3_^−^-responsive genes, such as *NRT2.1* and nitrate reductase 1 (*NIA1*), and their activity is modulated by NO_3_^−^ availability [25,26,27]. TGA factors bind to as-1-like elements adjacent to NREs, facilitating the assembly of enhanceosomes that synergistically boost transcriptional activation. The redox status of TGAs, modulated by thioredoxins, adds another layer of regulation linking N signaling to cellular energy state [25].

High-affinity nitrate transporters like NRT2.1 also participate in signaling, with their expression and stability tightly regulated by NO_3_^−^ and internal N status. NRT2.1 requires interaction with the partner protein NAR2.1 (NRT3.1) for plasma membrane localization and function [28,29]. Its degradation under N-sufficient conditions, mediated by the ubiquitin–proteasome system involving the E3 ligase nitrate-inducible btf3-like protein 1 (NBIP1), acts as a signal to repress LR growth under high N [30,31]. NBIP1 recognizes a degron motif in cytoplasmic loop of NRT2.1, ubiquitinating lysine residues to target it for 26S proteasomal degradation. This degradation serves as a metabolic rheostat: high cellular N sufficiency (e.g., glutamine accumulation) upregulates *NBIP1* expression, creating a negative feedback loop that curtails further N uptake and LR proliferation [32,33].

### 2.2. Ammonium Sensing: Transporters and Toxicity Responses

Ammonium (NH_4_^+^) sensing, while less understood mechanistically than NO_3_^−^ sensing, involves transporters like ammonium transporter 1;1 (AMT1;1). AMT1;1 undergoes phosphorylation-dependent regulation by CIPK23, analogous to NRT1.1, which controls its transport activity and, potentially, signaling outputs [34,35,36]. High NH_4_^+^ concentrations can induce ammonium toxicity, suppressing LR development through mechanisms involving pH imbalance, reactive oxygen species (ROS) accumulation, and disruption of hormone homeostasis, particularly auxin transport and signaling [37,38,39]. At the cellular level, NH_4_^+^ assimilation acidifies the cytosol, activating plasma membrane H^+^-ATPases to restore pH homeostasis. This ATP-dependent process depletes cellular energy, indirectly inhibiting energy-intensive processes like LR initiation. Concurrently, NH_4_^+^-induced ROS burst activates mitogen-activated protein kinase (MAPK) cascades that repress auxin transporter expression [40].

### 2.3. Organic N Sensing: Amino Acid Transporters and Signaling

Organic N forms, such as amino acids, are also perceived, often via specific transporters (e.g., lysine–histidine transporter 1 (LHT1), amino acid permeases (AAPs)), and can influence LR development, although their signaling pathways are less defined and may partially overlap with nitrate or ammonium signaling or involve specific peptide receptors [41,42,43]. For instance, glutamine uptake via AAP1 activates TOR kinase signaling which promotes root growth but may suppress LR initiation under high organic N by enhancing cytokinin sensitivity [44,45]. These primary sensing and signaling modules converge to regulate downstream transcriptional networks and hormonal pathways that directly impinge on LR developmental programs.

The three N-sensing modules (nitrate, ammonium, organic N) integrate via shared signaling nodes and nutrient status feedback. For example, high ammonium inhibits nitrate uptake by downregulating *NRT1.1* and *NRT2.1* [37], while nitrate represses *AMT1;1* expression via NLP7. Organic N (e.g., glutamine) activates target of rapamycin (TOR), which antagonizes NLP7-dependent nitrate responses, creating a priority hierarchy where organic N suppresses nitrate foraging [44]. Conversely, under N deficiency, all three modules synergize: NRT1.1 activates auxin pathways, AMT1;1 modulates pH to enhance nutrient availability, and amino acid transporters upregulate ARF19 to promote LR initiation [42]. This integration ensures that plants prioritize the most accessible N form while maintaining plasticity.

## 3. Integration of N Signals with Lateral Root Development: Hormonal Crosstalk and Transcriptional Networks

This section focuses on how N signals integrate with LR developmental programs through complex interactions between hormones (auxin, cytokinins, ethylene) and transcription factors (NLP7, ANR1), highlighting the multi-layered regulation of LR initiation, emergence, and elongation.

### 3.1. Hormonal Crosstalk

#### 3.1.1. Auxin

The core developmental pathway for LR formation is orchestrated by the phytohormone auxin. Auxin maxima, established through polar auxin transport (PAT) mediated by PIN-formed (PIN) efflux carriers (particularly PIN1, PIN3, PIN4, PIN7) and auxin-resistant 1/like AUX1 (AUX1/LAX) influx carriers within the pericycle and overlaying tissues, are essential for specifying founder cells and initiating LR formation [8,46,47]. This process begins when auxin accumulates in xylem-pole pericycle cells, activating the expression of cell cycle regulators like cyclin D3;1 (*CYCD3;1*) and triggering G2-to-M-phase transition in founder cells [48]. This auxin gradient activates the canonical signaling pathway: auxin promotes the degradation of auxin/indole-3-acetic acid (Aux/IAA) repressors via the Skp1–Cullin–F-box protein complex (SCF) ubiquitin ligase complex, releasing ARFs to activate transcription of genes like *LBD16/18/29*, which are critical for asymmetric cell divisions initiating LR formation [49,50,51,52]. LBD proteins induce periclinal divisions by activating GATA transcription factor 23 (GATA23) and suppressing endodermal suppressor genes, thereby defining the boundary between the developing primordium and surrounding tissues [53]. N signals profoundly intersect with this auxin pathway at multiple levels to modulate LR development.

Local nitrate availability exerts a major influence on auxin transport and sensitivity. NRT1.1 plays a pivotal role beyond initial sensing. Under low-nitrate conditions, unphosphorylated NRT1.1 facilitates auxin uptake into root tip cells, depleting auxin from the basal meristem and elongation zone, thereby indirectly inhibiting LR initiation and emergence in these regions [54,55]. Conversely, under higher local nitrate, phosphorylated NRT1.1 has reduced auxin transport capacity, allowing auxin to accumulate in the basal root regions, promoting LR initiation and emergence [15,56]. This switch creates a spatial auxin redistribution: high nitrate in the elongation zone stabilizes PIN1 in basal membranes, channeling auxin toward pericycle cells poised for LR initiation [57].

Furthermore, nitrate directly modulates the expression and localization of PIN auxin efflux carriers. Nitrate deprivation can repress *PIN1*, *PIN2*, and *PIN7* expression and alter PIN protein abundance or polarity, disrupting auxin transport and maxima formation necessary for LR development [58,59,60]. For example, low nitrate induces PIN2 internalization via clathrin-mediated endocytosis, reducing auxin reflux from the root apex and depleting basal auxin pools [61].

Nitrate also influences auxin biosynthesis. The tryptophan aminotransferase of *Arabidopsis* 2 (*TAR2*) gene encoding a key enzyme in the indole-3-pyruvic acid (IPyA) auxin biosynthesis pathway is strongly induced by low-N conditions in the pericycle and vasculature [62,63]. This localized auxin production contributes to LR initiation under N deficiency. The transcriptional regulators NLP6 and NLP7 directly bind to the promoter of *TAR2*, activating its expression in response to nitrate resupply after starvation, linking nitrate signaling to auxin biosynthesis [64]. NLP7 binds to two NRE motifs in the TAR2 promoter recruiting histone acetyltransferases that open chromatin to allow RNA polymerase II recruitment [65].

Downstream auxin signaling components are also N-regulated. The stability and activity of IAA28, an Aux/IAA repressor, are modulated by nitrate. Under high nitrate, IAA28 accumulates, repressing ARF activity and inhibiting LR initiation [66,67]. Nitrate deficiency promotes IAA28 degradation, releasing repression and facilitating LR development. The expression of *ARF8* and *ARF19* is also modulated by N availability, adding another layer of regulation [5,68]. Low N upregulates ARF19 via NLP7, creating a feed-forward loop that amplifies auxin signaling specifically in pericycle cells [69].

#### 3.1.2. Other Hormones

Beyond auxin, other hormones are critical integrators of N signals into LR development. Abscisic acid (ABA) accumulates under N deficiency and acts as a potent repressor of LR initiation and elongation. ABA signaling components like the pyrabactin resistance/pyr1-like (PYR/PYL) receptors and sucrose non-fermenting 1-related protein kinase 2 (SnRK2) kinases are involved in mediating N-dependent LR responses [70,71]. ABA antagonizes auxin transport and signaling, potentially explaining part of its inhibitory effect under low N. ABA induces expression of *IAA3*/SHY2 which stabilizes PIN proteins in an inactive conformation, reducing auxin efflux from the vasculature to the pericycle [72]. Conversely, ABA can also promote LR growth under specific contexts or moderate stress.

Cytokinins (CKs) are negative regulators of LR development whose biosynthesis and signaling are often upregulated by high N availability, particularly nitrate. The ISOPENTENYLTRANSFERASE (*IPT*) genes, encoding rate-limiting enzymes in CK biosynthesis, are induced by nitrate, leading to increased CK levels [73,74,75]. CKs act in the vasculature to suppress LR initiation, likely by inhibiting pericycle cell activation and promoting the expression of *IAA3*/*SHY2*, which stabilizes PIN proteins in an inactive configuration, reducing auxin transport to the pericycle [76,77]. CK-activated *Arabidopsis* response regulator 1 (ARR1) transcription factor directly represses *PIN1* and *PIN7* expression in protoxylem cells, blocking the auxin channel toward pericycle founder cells [78,79].

Ethylene biosynthesis is frequently enhanced under low-N or high-NH_4_^+^ conditions and generally promotes LR initiation and emergence. Ethylene can stimulate auxin biosynthesis and transport toward the root apex and pericycle, facilitating LR formation [80]. Ethylene stabilizes ethylene-insensitive 3/ein3-like 1 (EIN3/EIL1) transcription factors, which activate anthranilate synthase α subunit 1 (*ASA1*) and *TAR2* genes in the root apex, increasing auxin synthesis. Concurrently, ethylene inhibits PIN2-mediated auxin reflux, redirecting auxin flow toward the pericycle [81]. The interaction between ethylene and auxin is crucial for the LR response to N limitation.

Gibberellins (GAs) exhibit complex, context-dependent effects. GAs generally promote cell elongation. N deficiency can reduce bioactive GA levels, contributing to inhibited primary root growth. However, GA signaling also interacts with DELLA proteins, which are negative regulators integrating multiple signals (including N and other hormones) that can influence LR development [82]. Under low N, accumulated DELLAs sequester phytochrome-interacting factor (PIF) transcription factors, suppressing cell elongation in LRs. Conversely, GA-mediated DELLA degradation releases ARF6 from repression, promoting LR emergence [83].

Brassinosteroids (BRs) are emerging as critical regulators of LR development under low N stress. BR signaling, mediated by receptors like brassinosteroid-insensitive 1 (BRI1) and downstream kinases such as BR signaling kinase 3 (BSK3), modulates root foraging strategies in response to N limitation. Natural variation in *BSK3* expression has been shown to tune BR signaling intensity, with higher BSK3 activity enhancing LR elongation and branching in nitrate-poor environments [84]. BRs synergize with auxin by promoting PIN auxin transporter expression and stabilizing ARF transcription factors, thereby reinforcing auxin-dependent LR initiation under low N [85]. Conversely, excessive BR signaling may antagonize LR development by upregulating cytokinin biosynthesis, highlighting context-dependent crosstalk between BRs and other hormones in N-dependent root plasticity [86].

### 3.2. Transcriptional Networks

Specific transcription factors act as key nodal points integrating N status with the LR developmental machinery. The MADS-box transcription factor ANR1 was the first identified component specifically mediating the stimulatory effect of localized nitrate on LR elongation in *Arabidopsis* [12,87]. *ANR1* expression is induced by nitrate in the LR primordia. It promotes LR elongation likely by regulating cell cycle genes and potentially interacting with hormone pathways, although its direct targets in LR elongation remain less defined than its role in root foraging. The NLP family, especially NLP6, NLP7, and NLP8, comprises central integrators. As mentioned, they regulate *TAR2* and directly control genes involved in cell cycle progression and meristem activity (e.g., *CYCD3;1*), impacting both LR initiation and subsequent growth phases [69,88,89]. The SPL9 transcription factor, regulated by microRNA156 (miR156), is a repressor of LR development under high-N conditions. High N promotes the accumulation of mature SPL9 protein, which directly represses the expression of *LBD29*, a key activator of LR initiation downstream of auxin signaling [90,91]. Conversely, under low N, miR156 levels increase, repressing SPL9 and derepressing LBD29, allowing LR initiation. The teosinte branched 1/cycloidea/pcf 20 (TCP20) transcription factor interacts with NLP6/7 on the promoters of nitrate-responsive genes and has been implicated in regulating the proliferation of root meristems in response to nitrate, impacting LR growth potential [92,93]. The hypersensitive to low P and nitrate response 1/hrs1 homolog 1 (HRS1/HHO1) regulatory module plays a crucial role in suppressing LR initiation under high-nitrate conditions. Specifically, HRS1 functions as a transcriptional activator of HHO1, which subsequently mediates the inhibition of LR development (Table 1) [94,95].

## 4. Systemic N Signaling Coordinating Shoot–Root Communication

### 4.1. CEP Signaling

Plant N responses involve not only local root sensing but also systemic signaling of whole-plant N status, which critically coordinates root plasticity with shoot requirements. A major systemic pathway involves CEP signaling. Under N-deficient conditions, CEP precursor genes are transcriptionally upregulated in roots, particularly in the vasculature and LR tips [96,97,98]. Processed CEPs are secreted into the xylem and transported to the shoot. In the shoot, CEPs bind to leucine-rich repeat receptor kinases CEPR1 and CEPR2 localized in the phloem [99,100].

CEP-CEPR1 binding triggers autophosphorylation of the receptor kinase domain, initiating a phosphorylation cascade that culminates in MAPK activation. This pathway induces the expression of C-terminally encoded peptide downstream transcription factor 1/2 (*CEPD1/2*) genes in phloem companion cells [101]. CEPD1/2 proteins (12–15 kDa) lack canonical signaling domains but contain glutaredoxin-like motifs. They dimerize and move via plasmodesmata into sieve elements, traveling through the phloem to roots [102].

In roots, CEPD1/2 act as mobile signals that promote the expression of high-affinity nitrate transporters, primarily NRT2.1, enhancing nitrate uptake capacity. Crucially, the CEP-CEPR-CEPD pathway also modulates LR development. Systemic N deficiency, signaled via CEPs, promotes LR growth. This promotion involves CEPD-dependent regulation and likely interacts with auxin transport or sensitivity pathways in the root, although the precise molecular targets in LR development are still being elucidated [69,103,104]. Recent evidence suggests that CEPD1 interacts with NRT1.1 in the root endodermis, enhancing its nitrate-sensing capacity and auxin redistribution to pericycle cells [105].

### 4.2. Cytokinin Signaling

The shoot also exerts control over LR development through the regulation of cytokinin biosynthesis. High-shoot-N status, particularly high nitrate levels perceived in the shoot, induces the expression of *IPT* genes (*IPT3*, *IPT5*, *IPT7*) encoding cytokinin biosynthetic enzymes [106,107,108]. The resulting cytokinins are transported via the xylem to the roots, where they act to repress LR initiation, providing a systemic brake on root branching when shoot N is sufficient. Conversely, low-shoot-N status reduces shoot-derived cytokinin supply, alleviating repression and allowing increased LR proliferation.

### 4.3. Sugar Signaling

Sugars, primarily sucrose transported from source leaves via the phloem, not only are essential energy and carbon skeletons but also act as signaling molecules. Sucrose levels influence LR development, and its signaling interacts with N signaling. The C/N balance is a critical systemic integrator. Low-C status (e.g., low light, limiting photosynthesis) can suppress LR development even under N deficiency, while adequate sucrose can promote LR growth under low-N conditions [109,110,111].

Key transcription factors like hexokinase1 (HXK1) and TOR kinase are central sensors and integrators of sugar and energy status, which interact with N signaling pathways to regulate growth, including LR development [112,113,114,115]. HXK1 senses glucose in the cytosol and represses *LBD29* expression via unknown intermediaries, while TOR kinase phosphorylates E2Fa transcription factor to promote cell cycle progression in LR primordia under high-C/N conditions [113]. For instance, TOR kinase activity, promoted by sugars and amino acids, regulates protein synthesis and cell proliferation, impacting root meristem activity and LR growth. The coordinated action of peptide hormones (CEP), phytohormones (CK), and metabolic signals (sucrose, C/N ratio) ensures that LR development is attuned to the overall nutritional status and photosynthetic capacity of the plant.

A stepwise model of systemic N signaling is as follows: (1) under N deficiency, root vasculature and LR tips upregulate *CEP* genes, producing CEPs; (2) CEPs are transported via the xylem to shoot phloem, where they bind CEPR1/2 receptors; (3) CEPR1/2 activation induces *CEPD1/2* expression in phloem companion cells; (4) CEPD1/2 proteins move via the phloem to roots, upregulating NRT2.1 and promoting LR growth; (5) shoot-derived cytokinins (from IPT3/5/7 induction under high shoot N) counteract this by repressing LR initiation. This “root-to-shoot-to-root” loop coordinates local root foraging with whole-plant N status.

## 5. Modulatory Influences: C/N Balance, Epigenetics, and Environmental Interactions

### 5.1. C/N Balance: Metabolic Integrators of LR Plasticity

The regulation of LR development by N is further modulated by internal metabolic status and external environmental cues. The C/N balance serves as a fundamental metabolic integrator. Plants continuously monitor the ratio of C skeletons (primarily derived from photosynthesis) to reduced N compounds (amino acids, nucleotides) to coordinate growth and resource allocation. Disruption of this balance, such as high N with low C (e.g., low light), or low N with high C, triggers specific adaptive responses impacting RSA. Signaling pathways involving SnRK1, activated under energy deficit (low C/high energy demand), and TOR kinase, activated under energy sufficiency (high C/N), act antagonistically to regulate gene expression and cellular processes, including those in roots [112,115,116]. SnRK1 activation under low C/high N stress can promote catabolic processes and inhibit growth, potentially suppressing LR development, whereas TOR activation under high C/N promotes anabolic processes and growth. Under low-energy conditions, SnRK1 phosphorylates and activates key transcription factors including bZIP1. The bZIP1-NLP7 dimerization may modulate nitrate signaling output, thereby integrating C/N status with developmental processes like LR formation [117,118]. The glutamine synthetase/glutamate synthase GS/GOGAT cycle, central to ammonium assimilation, produces glutamine as a key nitrogen status signal. Glutamine levels correlate with C/N balance and can influence the expression of genes involved in LR development, potentially via modulating TOR activity or interacting with specific transcription factors [119,120].

### 5.2. Post-Translational and Epigenetic Regulation

Post-translational modifications (PTMs) provide rapid and reversible mechanisms to fine-tune the activity and stability of key N signaling and LR developmental components. Ubiquitination mediated by E3 ligases is crucial for targeted protein degradation. As mentioned, the E3 ligase NBIP1 ubiquitinates NRT2.1 under high N, leading to its degradation and contributing to the repression of LR growth [32,33]. Conversely, specific E3 ligases might target repressors of LR development under low N. Phosphorylation is ubiquitous in N signaling: phosphorylation of NRT1.1 by CIPK23 controls its affinity and signaling state; phosphorylation of NLP7 promotes its nuclear localization and activity; phosphorylation of PINs regulates their activity and subcellular trafficking, impacting auxin transport [121,122,123,124,125]; and SnRKs act downstream of N signals to phosphorylate targets like NLPs, TGAs, or components of hormone pathways.

Epigenetic regulation, including DNA methylation, histone modifications, and chromatin remodeling, offers a layer of stable or metastable control over gene expression programs in response to environmental cues like N availability. While less explored in the specific context of LR development compared to primary N responses, evidence suggests its involvement. Changes in histone methylation marks (e.g., H3K4me3 activation, H3K27me3 repression) and acetylation occur on promoters of N-responsive genes [126,127,128]. Mutations in chromatin remodelers (e.g., switch/sucrose non-fermentable (SWI/SNF) complexes) or histone modifiers can alter root growth responses to N [129,130]. Small RNAs, particularly microRNAs (miRNAs), play regulatory roles. As established, miR156 post-transcriptionally represses SPL9 by targeting its mRNA. Under N limitation, elevated miR156 levels reduce SPL9 accumulation, consequently promoting LR initiation [90,91]. Other miRNAs (e.g., miR393, miR167) regulating auxin signaling components are also modulated by N, potentially fine-tuning auxin responses in roots during N adaptation [131,132].

### 5.3. Interactions with Other Nutrients and Environmental Factors

LR responses to N do not occur in isolation but are integrated with responses to other nutrients and environmental factors. P deficiency strongly stimulates LR proliferation and root hair development. Complex interactions exist: P deficiency can override or modulate N responses. For instance, P starvation often induces genes involved in N metabolism and can alter LR responses to localized N patches [133,134]. S deficiency also impacts RSA and interacts with N signaling, partly through shared metabolites like glutathione and regulatory overlaps [135,136]. Soil pH, influenced by N form (NH_4_^+^ acidification, NO_3_^−^ alkalinization), significantly affects nutrient availability, rhizosphere microbiome composition, and root growth directly [137,138]. Microbial interactions in the rhizosphere, including associations with N-fixing bacteria or mycorrhizal fungi, profoundly alter RSA and N acquisition pathways, often stimulating LR formation to support symbiosis interfaces [139,140,141]. Water availability (drought/flooding) modulates N responses: drought inhibits LR growth, and partial rootzone drying coordinates with N placement strategies, while flooding-induced hypoxia strongly suppresses LR development and reprograms N metabolism [142,143].

## 6. Emerging Insights and Future Perspectives

### 6.1. Emerging Insights

Recent technological advances are providing unprecedented resolution in the spatiotemporal dynamics and cellular heterogeneity of N responses in roots. Single-cell and spatial transcriptomics are revealing distinct gene expression programs in different root cell types (pericycle, endodermis, cortex, epidermis, vasculature) in response to N availability or form [144]. For instance, xylem-pole pericycle cells (LR founder cells) exhibit unique upregulation of *AUX1* and *CYCD3;1* under low N, while phloem cells prioritize *CEP* expression for systemic signaling [145,146]. Spatial transcriptomics further maps these responses to specific root zones: LR primordia in the maturation zone show enriched *NLP7* and *LBD16* expression, whereas the root apex prioritizes *NRT2.1* [147]. These data challenge the traditional view of uniform root responses, revealing a “division of labor” where distinct cell types handle local sensing, signal transduction, or systemic communication. Such granularity is critical for designing precise genetic interventions—e.g., targeting NLP7 specifically in pericycle cells to enhance LR initiation without disrupting other root functions.

Live-cell imaging combined with advanced microscopy (confocal, light sheet) and biosensors for ions (Ca^2+^, pH), hormones (auxin, cytokinin), and redox status (ROS, glutathione) allows real-time visualization of signaling events and developmental dynamics in intact roots under varying N conditions [148,149]. This reveals the kinetics of auxin maxima formation, Ca^2+^ waves, ROS bursts, and cell division patterns during LR initiation and emergence in response to localized N sources or systemic cues. Computational modeling integrating molecular interaction networks, hormone fluxes, and biophysical constraints is becoming increasingly sophisticated, allowing predictions of RSA plasticity under complex N regimes and facilitating the design of targeted interventions [150,151].

### 6.2. Future Perspectives

Despite significant progress, critical knowledge gaps remain. While major transceptors like NRT1.1 are established, the precise molecular mechanisms of initial N perception, particularly for ammonium and organic N, require further elucidation. The nature of the putative nitrate “receptor” upstream of calcium signaling, distinct from NRT1.1 transport/signaling, is still debated [152,153]. Additionally, ammonium sensing mechanisms are poorly defined—AMT1;1 is proposed as an ammonium transceptor [34], but its direct role in signaling (vs. transport) is debated [36]. The downstream targets of key transcription factors like ANR1 in promoting LR elongation are still incompletely mapped. The exact molecular mechanisms by which systemic signals like CEPDs modulate LR development need definition. How C/N signaling nodes like SnRK1 and TOR precisely interface with the core auxin machinery and N-responsive transcription factors to gate LR initiation or elongation requires deeper investigation. The role of epigenetic memory in long-term adaptation of root systems to fluctuating N regimes is largely unexplored.

Translating mechanistic knowledge from model systems like *Arabidopsis* to major crops, which often have more complex root systems and different N response dynamics (e.g., cereals with seminal and crown roots), presents a significant challenge but is essential for agricultural impact [154,155,156,157]. In rice, overexpression of *OsNLP* enhances LR density and N uptake efficiency under low N [158], while editing *OsSPL* increases LR branching in nitrate-poor soils [159]. In maize, *ZmANR1* orthologs are associated with enhanced root foraging in N patches [160]. These examples demonstrate that conserved regulators (NLP, SPL, ANR1) can be targeted across species, but crop-specific adjustments are needed—e.g., accounting for brace roots of maize or aerenchyma of rice. Integrating such species-specific traits with conserved N signaling modules will accelerate the development of N-efficient crops. Future research must bridge fundamental discoveries in model systems with applied crop physiology and breeding, leveraging genomics, phenomics, and gene editing technologies to develop next-generation crops with inherently superior N foraging capabilities.

## 7. Conclusions

N exerts a profound and multi-layered influence on lateral root development, a cornerstone of root system plasticity. This review has synthesized the complex journey from initial N perception at the root surface, primarily via transceptors like NRT1.1 for nitrate, through intricate intracellular signaling cascades involving calcium fluxes, phosphorylation events, and master transcriptional regulators like NLP7 and TGA factors. These pathways converge to modulate the core auxin-dependent LR developmental machinery, impacting PIN-mediated auxin transport, Aux/IAA-ARF dynamics, and key transcription factors like LBDs and ANR1 at specific developmental stages (initiation, emergence, elongation). Systemic signals, notably the CEP-CEPR-CEPD peptide relay conveying root N deficiency to the shoot and shoot-derived cytokinins communicating shoot N sufficiency back to the roots, integrate whole-plant N status and carbon availability (C/N balance) with local root responses. This regulatory network is further fine-tuned by post-translational modifications (ubiquitination, phosphorylation), epigenetic mechanisms, and continuous interactions with other nutrients (P, S), soil properties (pH), and the rhizosphere microbiome. The emergence of single-cell technologies and advanced imaging is revealing unprecedented cellular heterogeneity in N responses. While significant progress has been made, critical gaps remain in understanding specific receptor mechanisms, defining complete signaling pathways for different N forms, mapping transcription factor targets comprehensively, and deciphering the epigenetic code of N memory. Translating this fundamental knowledge into strategies for breeding or engineering crops with optimized root architectures for enhanced N acquisition efficiency represents the critical next frontier, offering a pathway toward more sustainable and productive agriculture in the face of global challenges.

A proposed model integrating N-dependent LR regulation is as follows: (1) local nitrate is sensed by NRT1.1, which modulates auxin transport and activates NLP7 nuclear translocation; (2) NLP7 and TGA factors coordinate TAR2 and CYCD3;1 to promote LR initiation; (3) systemic signals (CEPs from roots, cytokinins from shoots) adjust LR growth based on whole-plant N status; (4) hormonal crosstalk (auxin–BR–cytokinin) and C/N balance (via TOR/SnRK1) fine-tune LR plasticity.

## Figures and Tables

**Table 1:** Key transcription factors integrating N signals into LR development.

Transcription Factor	Family	Nitrogen Regulation	Function in LR Development	Molecular Mechanism/Targets	Developmental Stage	Key References
NLP7	NIN-like protein	Activated by nitrate; nuclear translocation under high NO_3_^−^	Central integrator of nitrate signaling; promotes LR initiation and growth	Binds NRE motifs; activates *TAR2* (auxin biosynthesis) and *CYCD3;1* (cell cycle); interacts with TGA factors	Initiation and primordium	[18,19,20,64,69]
ANR1	MADS-box	Induced by localized nitrate	Mediates nitrate-stimulated LR elongation	Regulates cell cycle genes; potential interaction with auxin pathways	Elongation	[12,87]
SPL9	SQUAMOSA promoter-binding protein-like	Repressed by miR156 under low N; accumulated under high N	Represses LR initiation under high-N conditions	Directly represses *LBD29* (auxin signaling activator)	Initiation	[90,91]
TGA1/TGA4	bZIP	Activated by nitrate sensing	Enhances nitrate-responsive gene expression; cooperates with NLP7	Forms complexes with NLP7; binds as-1-like elements; regulates *NRT2.1* and *NIA1*	Initiation and signaling	[25,27]
TCP20	TCP	Interacts with N availability signals	Regulates root meristem proliferation in response to nitrate	Interacts with NLP6/7 on promoters of nitrate-responsive genes	Growth potential	[92,93]
HRS1/HHO1	G2-like	Induced under high nitrate	Suppresses LR initiation under high N	HRS1 activates *HHO1* expression; HHO1 mediates LR inhibition	Initiation	[94,95]
LBD16/18/29	LBD/ASL	Activated by ARF7/ARF19 under low N	Direct regulators of asymmetric cell division in LR founder cells	Induced by auxin; activate *GATA23*; suppress endodermal barriers	Initiation and primordium	[49,50,52]

## Data Availability

Not applicable.
